# The Improvement of Sumatran Elephant (*Elephas maximus* *sumatranus*) Dung Particleboard Characteristics Using Bamboo Layering

**DOI:** 10.3390/polym14163330

**Published:** 2022-08-16

**Authors:** Rudi Hartono, Apri Heri Iswanto, Evalina Herawati, Reski Eka Suramana, Jajang Sutiawan, Yusup Amin, Ihak Sumardi

**Affiliations:** 1 Department of Forest Products, Faculty of Forestry, Universitas Sumatera Utara, Medan 20155, Indonesia; 2 Research Center for Biomass and Bioproducts, National Research and Innovation Agency, Cibinong 16911, Indonesia; 3 School of Life Sciences and Technology, Institut Teknologi Bandung, Bandung 40132, Indonesia

**Keywords:** bamboo, elephant dung, layering, particleboard

## Abstract

The use of natural fibers or particles as alternative raw materials for particleboard production is essential due to the shrinking forest area. Currently, dung waste from the Sumatran elephant (*Elephas maximus sumatranus*) is being used as a raw material for particleboard due to its high fiber content. Although the product still has inferior mechanical and physical characteristics, it can be improved by layering bamboo. Therefore, this study aimed to enhance the mechanical and physical qualities of elephant dung particleboard by adding layers of bamboo. The particleboard constructed had three layers; namely, the face and back in the form of a bamboo layers, as well as the core, which was in the form of elephant dung. The elephant dung was evenly mixed with isocyanate adhesive using a spray gun, and the bamboo layers were coated with adhesive on one side of the surface. The sample was subjected to a hot press at a temperature of 150 °C and 30 kg/cm^2^ pressure for 10 min. Generally, JIS A 5908-2003 is the specification used to test the physical and mechanical properties of particleboard. During the experiment, the characteristics examined include density, moisture content, water absorption, thickness swelling, modulus of elasticity, modulus of rupture, and internal bonding, which were enhanced by using layers of bamboo. The results showed that the physical properties of the particleboard with bamboo layers were a density of 0.62–0.69 g/cm^3^, a moisture content of 7.87–10.35%, water absorption of 38.27–68.58%, and a thickness swelling of 10.87–30.00%, which met the minimum standards of JIS A 5908-2003. The mechanical characteristics had values for the modulus of elasticity of 1952–7282 MPa, the modulus of rupture of 20.44–68.27 MPa, and the internal bonding of 0.16–0.38 MPa, which met the JIS A 5908-2003 standard. Based on these results, the particleboard with Belangke bamboo layers was the best in this study.

## 1. Introduction

In 2020, the production and consumption of wood-based panels decreased by 3.3% and 4.3%, respectively, due to the COVID-19 pandemic [1]. The number of panels used for structural purposes was also reduced by 2.2%, while the amount of nonstructural uses fell by 5.6% [1]. Moreover, the FAO anticipates that the manufacturing of wood-based panels will rise after the pandemic subsides in 2022 [1]. This will lead to an increase in the use of raw materials such as particleboard to make wood-based panels.

The use of natural fibers or particles as substitute raw materials for particleboard production is essential due to the shrinking forest area [2]. Particleboard has been created using a variety of natural fibers as raw materials, which include rice husks, sugarcane bagasse, corn stalks, nipah and salacca fronds, kenaf, and sorghum [3,4,5,6,7,8,9,10,11]. The ability to address environmental issues, and thereby have an impact on ecological and socioeconomic elements, are the two benefits of using natural fibers in the production of particleboard [12]. Azevedo et al. [13] also suggested that a substitute for the proper management of natural fibers is the use and development of natural fiber materials in composite raw materials.

One abundant substance containing natural fibers that has not been used is Sumatran elephant dung waste (*Elephas maximus sumatranus*). This waste has the potential to be used as a raw material for particleboard due to its high fiber content. As a ruminant mammal, the Sumatran elephant only absorbs 40% of its nutritional intake [14], and therefore has been employed as a biogas producer that can create up to 100–130 kg of manure daily [15]. Elephant dung waste was successfully used by Farah et al. [16] to create unusual paper and as a primary component of particleboard [17,18].

Jati et al. [17] successfully produced particleboard from elephant dung with a density of 0.8 kg/cm^3^ using a 10% citric acid adhesive. Although the product complied with the JIS A 5908-2003 requirements for modulus of elasticity and rupture, the particleboard’s internal bonding and dimensional stability did not fulfill the standard. To overcome these issues, Widyorini et al. [18] improved the pressing temperature and citric acid content, which significantly increased the dimensional stability of the particleboard generated from elephant dung fibers. In this investigation, a 200 °C pressing temperature and a 20% citric acid concentration were ideal.

Hartono et al. [19] also used the addition of wood shavings to enhance the physical and mechanical characteristics of particleboard made from elephant dung. It was discovered that the elephant dung particleboard’s physical and mechanical qualities, as well as its moisture content and water absorption, were considerably changed by the addition of wood shavings. For this experiment, a 50/50 ratio of wood shavings to elephant dung was ideal, which met the JIS A 5908-2003 requirements except for thickness swelling.

Another effort with the potential to improve the physical and mechanical properties of particleboard is by using bamboo layers. According to Iswanto et al. [20], overlaying sorghum bagasse particleboard allowed for an increase in the material’s modulus of rupture and elasticity, which had the highest value when using bamboo strands. In addition, Sari and Mora [21], Umam et al. [22], Nemli and Çolakoǧlu [23], and Barbosa et al. [24] reported that bamboo layers significantly improved the physical and mechanical properties of particleboard. Therefore, this study aimed to improve the mechanical and physical qualities of elephant dung particleboard by adding layers of bamboo.

## 2. Materials and Methods

### 2.1. Materials

The materials used in this study were elephant (*Elephas maximus sumatranus*) dung, Belangke bamboo (*Gigantochloa pruriens*), Betung bamboo (*Dendrocalamus asper*), Tali bamboo (*Gigantochloa apus*), Kuning bamboo (*Bambusa vulgaris*), and Talang bamboo (*Schizostachyum brachycladum*). In addition, an isocyanate adhesive with a content of 7% and solid content of 98% was used.

The manufacturing of particleboard began with the washing of the elephant dung to collect the fiber. Subsequently, the fiber was air-dried and the elephant dung was oven-dried for 24 h at 103 °C until it reached a moisture content of 8%. Belangke, Betung, Tali, Kuning, and Talang bamboo were cut to a length of 20 cm, a width of 1 cm, and a thickness of 1 mm. The bamboo was dried in an oven set at 103 °C for 24 h until it had an 8% moisture content.

### 2.2. Fabrication and Testing of Particleboard 

**Figure 1:**
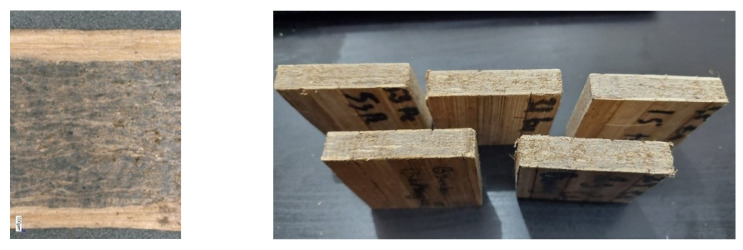
Sample arrangement.

The particleboard in this study has 3 layers: the face and back in the form of bamboo layers and the core in the form of elephant dung, as shown in Figure 1. The elephant dung was evenly mixed with isocyanate adhesive using a spray gun, and the bamboo layers were coated with adhesive on one side of the surface (Table 1). Subsequently, the elephant dung and bamboo layers were arranged using the face, core, and back, and were put into molds measuring 20 cm (length) and 20 cm (width). The sample was pressed using a hot press at a temperature of 150 °C for 10 min and a pressure of 30 kg/cm^2^, followed by 14-day conditioning at room temperature (20–25 °C). The JIS A 5908-2003 standard was used for testing the mechanical and physical characteristics of particleboard [25]. In this study, the physical and mechanical characteristics examined included density, moisture content, water absorption, thickness swelling, modulus of elasticity, modulus of rupture, and internal bonding.

### 2.3. Morphology Analysis

A latest-generation KEYENCE VHX 6000 digital microscope (Mechelen, Belgium), with an exceptional depth of focus and the ability to perform measurements and analyze living and nonliving materials, was used. This microscope had high-quality digital image recording capabilities provided by components such as the VH-Z20T (Osaka, Japan) ultra-small, high-performance zoom lens (20–200× magnification) and the VH-Z250T dual-light, high-magnification zoom lens (250–2500× magnification). The glue lines formed in the elephant dung particleboard layered with Belangke bamboo and the adhesive distribution of the core layer were analyzed using the KEYENCE VHX 6000 digital microscope at 200× magnification. In addition, a Quattro S field-emission scanning electron microscope (FE-SEM, Thermo Scientific, Vlastimila Pecha, Czech Republic.) was utilized for the analysis of references.

### 2.4. Data Analysis

A simple, completely randomized design (CRD) was used by analyzing a single factor as outlined in previous studies [10]. The factors determined were treatments for bamboo layers; namely, without layering bamboo (control) and with layering of Belangke, Tali, Betung, Kuning, or Talang bamboo. Each treatment was repeated 3 times, and the data were analyzed using a 95% confidence interval. When the significance value was less than 0.05, the results obtained were significantly different and continued using Duncan’s further test.

## 3. Results and Discussions

### 3.1. Physical Properties

**Figure 2:**
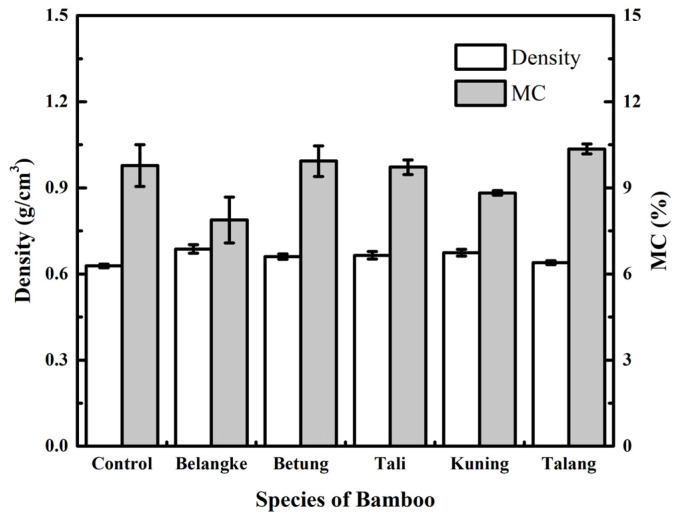
Density and moisture content (MC) of particleboards.

Figure 2 shows that the use of bamboo as a layering material for particleboard from elephant dung could lead to an increase in density. Rita et al. [26], Umam et al. [22], and Santoso et al. [27] reported that the addition of bamboo was able to increase the density of particleboard. Iswanto et al. [20] also stated that veneer and bamboo layers enhanced the density of sorghum bagasse and commercial particleboards [23,28]. Based on the results, the particleboard with a bamboo layer had a higher density than the control. The density of Betung bamboo is 0.78 g/cm^3^, while that of Tali is 0.93 g/cm^3^ [29], and that of elephant dung fiber is 0.11 g/cm^3^ [18]. Iswanto et al. [30] reported that the specific gravity value of Belangke bamboo ranged from 0.58 to 0.60. Therefore, it was used as a potential layering material for particleboard production. According to Xu et al. [31], the density of the raw materials used to construct particleboard significantly impacted the density of the finished product.

The results of the particleboard density measurements, which ranged from 0.4 to 0.9 g/cm^3^, met the JIS A 5908-2003 standard. However, they did not meet the density target with a value of 0.8 g/cm^3^ because the average board experienced a springback of 44%. According to the statistical evaluation, the bamboo layers significantly affected the particleboard density at a 95% confidence level. The results of Duncan’s further test showed that the particleboard layered with Belangke bamboo had the highest density.

Furthermore, Figure 2 shows the effects of the use of bamboo layers in particleboard made with elephant dung on the moisture content. At a 95% confidence level, the statistical analysis revealed that the bamboo layers significantly affected the moisture content of the particleboard. The results of Duncan’s further test showed that the highest moisture content was found in the particleboard with Talang bamboo layers (10.35%), while the lowest was for Belangke bamboo (7.88%). The JIS A 5908-2003 standard was met by the particleboard moisture content, with a maximum of 13%. This was in line with an earlier investigation that found that composite boards with a veneer contained less moisture than those without a veneer [32]. 

**Figure 3:**
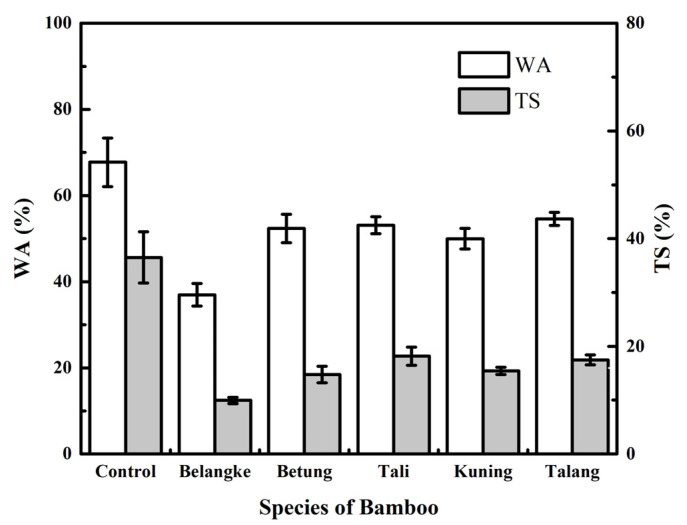
Water absorption (WA) and thickness swelling (TS) of particleboards.

Figure 3 shows the water absorption of the particleboard created for this investigation. It was discovered that the use of bamboo as a layering material in particleboard made from elephant dung decreased the water absorption of the particleboard and had a statistical effect. Based on the results of Duncan’s further test, the highest water absorption was discovered in the control (67.74%), while the lowest was in the particleboard constructed using Belangke bamboo layers (36.96%). Furthermore, the content of the total acid-insoluble lignin, holocellulose, alpha-cellulose, and extractive solubility in ethanol benzene (1:2) of the Belangke bamboo ranged from 25.25 to 27.56%, 63.56 to 66.66%, 39.70 to 44.40%, and 2.18 to 4.01%, respectively [30]. However, the elephant dung fiber had alpha-cellulose and acid-insoluble lignin of 71.67% and 30.50%, respectively [16]. The high alpha-cellulose content in elephant dung fiber made the control more hygroscopic than the particleboard constructed using Belangke bamboo layers. According to previous investigations, the chemical composition of raw materials influenced the chemical bonds that held particles to adhesives during manufacture, as well as the values for particleboard’s water absorption and thickness swelling [33,34]. This was in line with a study on particleboard with a rattan woven layer by Meranti, which reported that the addition of layers in the particleboard reduced the water absorption [35]. Similarly, it was also reported that water absorption could be reduced by using veneer layering [36,37].

Figure 3 also shows that the use of bamboo as layers in the particleboard made from elephant dung led to a decrease in the thickness swelling that was statistically significant. Based on Duncan’s further test, the highest thickness swelling was found in the control particleboard (36.50%), while the lowest thickness swelling was found in the particleboard with Belangke bamboo layers (9.95%). According to the JIS A 5908-2003 standard, only the particleboard with Belangke bamboo layers met the standard, with a maximum value of 12%.

### 3.2. Mechanical Properties

**Figure 4:**
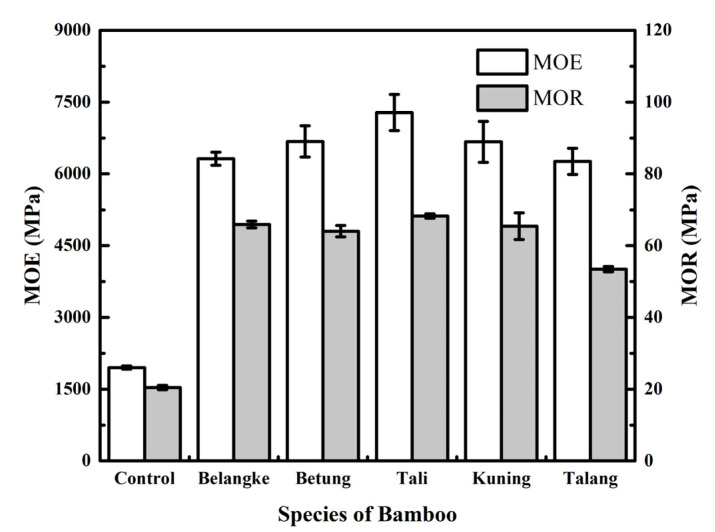
Modulus of elasticity (MOE) and modulus of rupture (MOR) of particleboards.

As shown in Figure 4, the modulus of elasticity (MOE) of the particleboard in this study had an average range of 1952–7282 MPa. This showed that the use of bamboo as a layered material could increase the MOE of the particleboard made from elephant dung. The increase in MOE in the particleboard was 320.70–373.08% that of the control. Umam et al. [22] reported that the addition of materials in the layer of bamboo and veneer could improve the MOE value. Iswanto et al. [30] also stated that layers of Belangke bamboo significantly improved the mechanical properties of the particleboards, thereby increasing the MOE and MOR values by 16 and 3 times, respectively. This was because the bamboo had good mechanical properties, with compressive, shear, and tensile strengths of 42.19 MPa, 7.63 MPa, and 163.8 MPa, respectively [30]. The results of the statistical analysis showed that the bamboo layers influenced the MOE of the particleboard. Furthermore, Duncan’s further test showed that the highest MOE was found in the Tali bamboo particleboard, while the lowest was in the control. All MOEs of the particleboards complied with the JIS A 5908-2003 standard, with a minimum value of 2040 MPa except for the control.

The modulus of rupture (MOR) of the particleboards had an average range of 20.49–68.27 MPa, as presented in Figure 4. Similar to the MOE, the use of bamboo as a layering material for particleboard of elephant dung led to an increase in the MOR value of the particleboards, which ranged from 261.46 to 333.89%. The highest MOR was for the Tali bamboo particleboard and the lowest was for the control, which was statistically significant. Iswanto et al. [20] reported that layering bamboo and veneer increased the mechanical properties of particleboard made from sorghum bagasse. It was also discovered that a veneer could improve the mechanical properties of particleboard made from cardboard, oil palm trunks, and empty fruit bunches [38,39]. All of the MORs for particleboards in this study met the JIS A 5908-2003 standard, with a minimum of 8.2 MPa.

**Figure 5:**
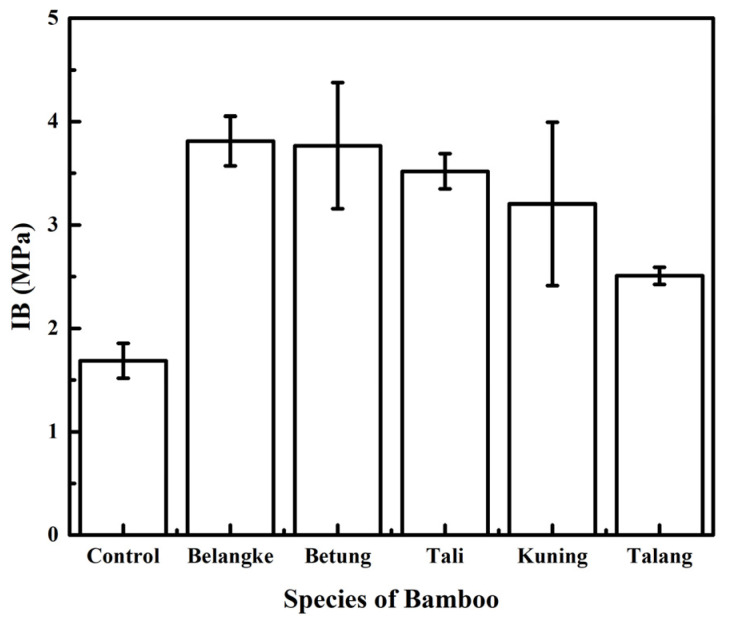
Internal bonding (IB) of particleboards.

Figure 5 shows the internal bonding (IB) of the particleboards, with an average value ranging from 0.16 to 0.38 MPa. Based on the results, the use of bamboo as a layering material in particleboard made from elephant dung led to an increase in the IB value. The highest IB was for the particleboard made with Belangke bamboo layers, while the lowest was for the control particleboard, which was statistically significant. The increase was caused by the high density of the bamboo, which affected the IB of the particleboard. The difference in IB values in particleboard layering was influenced by the anatomy of the bamboo layers. Furthermore, the differences in fiber size, lumen dimensions, and fiber wall thickness affected the quality class of the bamboo [40]. The IBs of the particleboards in this study met the minimum value of 0.15 MPa, as stated in the JIS A 5908-2003. 

### 3.3. Morphology Analysis

**Figure 6:**
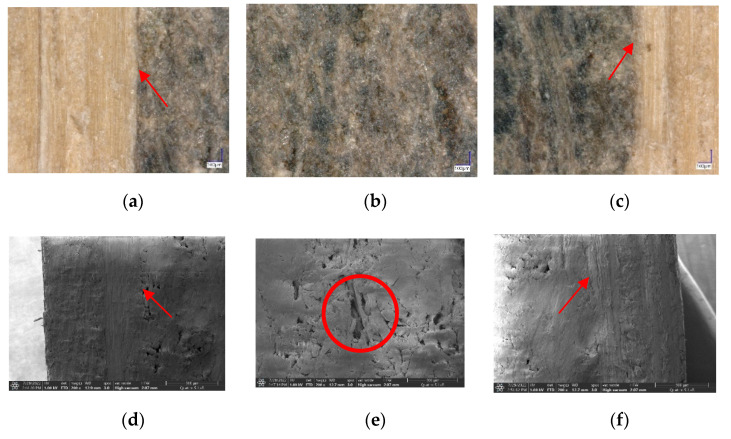
Morphology analysis of elephant dung particleboard layered with Belangke bamboo using KEYENCE VHX 6000 Digital Microscope and FE-SEM at 200× magnification: (**a**) face; (**b**) core; (**c**) back; (**d**) face; (**e**) core; (**f**) back.

Figure 6 shows the results of the morphological analysis using the KEYENCE VHX 6000 Digital Microscope and FE-SEM. The glue lines formed in the elephant dung particleboard layered with the Belangke bamboo were also identified in the face (Figure 6a,d) and back (Figure 6c,f) layers, which gave the product good adhesion. However, Figure 6e reveals that the core layer of the elephant dung particleboard had void spaces in its structure. Therefore, the dimensional stability of the particleboard constructed with Belangke bamboo layers was better than that of the control.

## 4. Conclusions

The use of bamboo layers improved the mechanical and physical characteristics of particleboard made from elephant dung. Based on the results, the physical properties of the elephant dung particleboard with bamboo layers had a density of 0.62–0.69 g/cm^3^, a moisture content of 7.87–10.35%, water absorption of 38.27–68.58%, and thickness swelling of 10.87–30.00%, and thus met the minimum standards of JIS A 5908-2003. The mechanical characteristics included modulus of elasticity (MOE) values of 1952–7282 MPa, modulus of rupture (MOR) values of 20.44–68.27 MPa, and internal bonding (IB) values of 0.16–0.38 MPa, which met the JIS A 5908-2003 standard. This indicated that the addition of Belangke bamboo layers resulted in the best particleboard in this study.

## Figures and Tables

**Table 1:** Particleboard raw material requirements.

Composition of Elephant Dung	Species of Bamboo	Isocyanate Content	Materials		
			Elephant Dung (g)	Isocyanate for Elephant Dung (g)	Isocyanate for Bamboo (g)
100%	-	7%	322.99	21.36	0
80%	Belangke	7%	258.39	17.08	4.27
80%	Tali	7%	258.39	17.08	4.27
80%	Petung	7%	258.39	17.08	4.27
80%	Kuning	7%	258.39	17.08	4.27
80%	Talang	7%	258.39	17.08	4.27

## Data Availability

The data presented in this study are available upon request from the corresponding author.
